# TRAF6 is a novel NS3-interacting protein that inhibits classical swine fever virus replication

**DOI:** 10.1038/s41598-017-06934-1

**Published:** 2017-07-27

**Authors:** Huifang Lv, Wang Dong, Zhi Cao, Xiaomeng Li, Jie Wang, Gui Qian, Qizhuang Lv, Chengbao Wang, Kangkang Guo, Yanming Zhang

**Affiliations:** 1 College of Veterinary Medicine, Northwest A&F University, No. 22 Xinong Road, Yangling, 712100 Shaanxi China; 2 Qingdao Yebio Biological Engineering Co., Ltd. (Yebio), No. 21 Aodongnan Road, Qingdao, 266114 Shandong China; 3 College of Biology & Pharmacy, Yulin Normal University, No. 1303 Jiaoyu East Road, Yulin, 537000 Guangxi China

## Abstract

Classical swine fever virus (CSFV) non-structural protein 3 (NS3) is a multifunctional non-structural protein that plays a major role in viral replication. However, how exactly NS3 exerts these functions remains unknown. Here, we identified tumour necrosis factor receptor-associated factor 6 (TRAF6) as a novel NS3-interacting protein via yeast two-hybrid analysis, co-immunoprecipitation, and glutathione *S*-transferase pull-down assays. Furthermore, we observed that TRAF6 overexpression significantly inhibited CSFV replication, and TRAF6 knockdown promoted CSFV replication in porcine alveolar macrophages. Additionally, TRAF6 was degraded during CSFV infection or NS3 expression exclusively, indicating that CSFV and TRAF6 were mutually antagonistic and that TRAF6 degradation might contribute to persistent CSFV replication. Moreover, nuclear factor-kappa B (NF-κB) activity and interferon (IFN)-β and interleukin (IL)-6 expression were increased in TRAF6-overexpressing cells, whereas TRAF6-knockdown cells exhibited decreased NF-κB activity and IFN-β and IL-6 levels. Notably, TRAF6 overexpression did not reduce CSFV replication following inhibition of NF-κB activation by p65 knockdown. Our findings revealed that TRAF6 inhibits CSFV replication via activation of NF-κB-signalling pathways along with increases in the expression of its targets IFN-β and IL-6. This work addresses a novel aspect concerning the regulation of innate antiviral immune response during CSFV infection.

## Introduction

Classical swine fever is a highly contagious viral disease caused by classical swine fever virus (CSFV), resulting in high mortality rates and causing tremendous economic losses in the swine industry. CSFV is a positive-stranded RNA virus with a 12.3 kb genome that encodes a single polyprotein that is co- and post-translationally processed into 12 mature proteins, including four structural proteins (C, E^rns^, E1, and E2) and eight non-structural (NS) proteins (N^pro^, P7, NS2, NS3, NS4A, NS4B, NS5A, and NS5B)^1^. CSFV belongs to the *Pestivirus* genus within the *Flaviviridae* family that includes the hepatitis C virus (HCV)^2^.

CSFV NS3 is a multifunctional non-structural protein that plays a major role in viral replication. NS3 is a chymotrypsin-like serine protease that utilizes NS4A as an essential cofactor^3, 4^. Aside from its serine-protease activity, NS3 possesses helicase and NTPase activities^5, 6^, with autocatalytic cleavage within the CSFV NS3 domain resulting in functional separation of protease and helicase activities^7^. NS3 helicase/NTPase activities are modulated by the NS3 protease domain and NS5B^8^, with CSFV NS3 interacting with NS5B to enhance RNA-dependent RNA polymerase activity, which can occur through NS3 binding to the two terminal sequences of NS5B through an NS3-protease domain^9, 10^. Additionally, NS3 is an internal ribosome entry site (IRES)-binding protein that increases IRES-dependent translation; however, CSFV NS5A and NS5B can reduce NS3-IRES interactions by competitively binding to the same sites in IRES-containing RNA sequences. The inhibitory effect of NS5B on NS3-IRES binding results from NS3-NS5B interactions^11^. Additionally, NS3 accumulation is related to the cytopathic effect (CPE) of CSFV^12^.

Tumour necrosis factor (TNF) receptor-associated factor 6 (TRAF6) is an essential adaptor protein common to the interleukin (IL)-1 receptor (IL-1R)/toll-like receptor (TLR) family and TNF-receptor superfamily. TRAF6 contains N-terminal Really Interesting New Gene (RING) and zinc-finger domains that enable its functioning as an ubiquitin E3 ligase essential for activation of downstream signalling cascades. TRAF6 also contains a coil-coil TRAF-N domain and a highly conserved TRAF-C domain, which contribute to their homo- and hetero-oligomerization and interactions with receptors and intracellular signalling proteins^13, 14^. Furthermore, TRAF6 is a critically important adaptor protein involved in the nuclear factor kappa-B (NF-κB)-signalling pathway. When ligand or stimulator, such as poly (I:C) or lipopolysaccharide (LPS), is added, TLR recruits adaptor proteins, including MyD88, TLR/IL-1R-domain containing adaptor inducing interferon-beta, and TRAF6. Moreover, lysine 63 (K63)-linked polyubiquitin chains are catalytically synthesized by ubiquitin ligase in the TRAF6 RING domain. K63-polyubiquitination targets TRAF6, and ubiquitinated TRAF6 initiates signalling cascades^15, 16^ that ultimately promote the rapid translocation of NF-κB into the nucleus, followed by phosphorylation of NF-κB p65 and transcriptional activation of various target genes, such as type 1 interferon (IFN) and inflammatory cytokines^17–19^. Previous studies showed that CSFV fails to activate the NF-κB-signalling pathway and decreases IFN-β and IL-6 levels^20–22^. In HCV, depletion of TRAF6 by HCV suppresses activation of NF-κB and induction of proinflammatory cytokines and enhances HCV replication^23^. We hypothesized that TRAF6 might affect CSFV replication by regulating the NF-κB-signalling pathway.

Most studies of CSFV NS3 focus on its protease, helicase, and NTPase activities; however, investigations of CSFV NS3-interacting host proteins and their impact on CSFV replication are limited. In this study, we demonstrated that CSFV NS3 interacted with TRAF6 and degraded TRAF6 to promote CSFV replication via the NF-κB-signalling pathway.

## Results

### Screening for cellular CSFV NS3-interacting proteins

**Figure 1:**
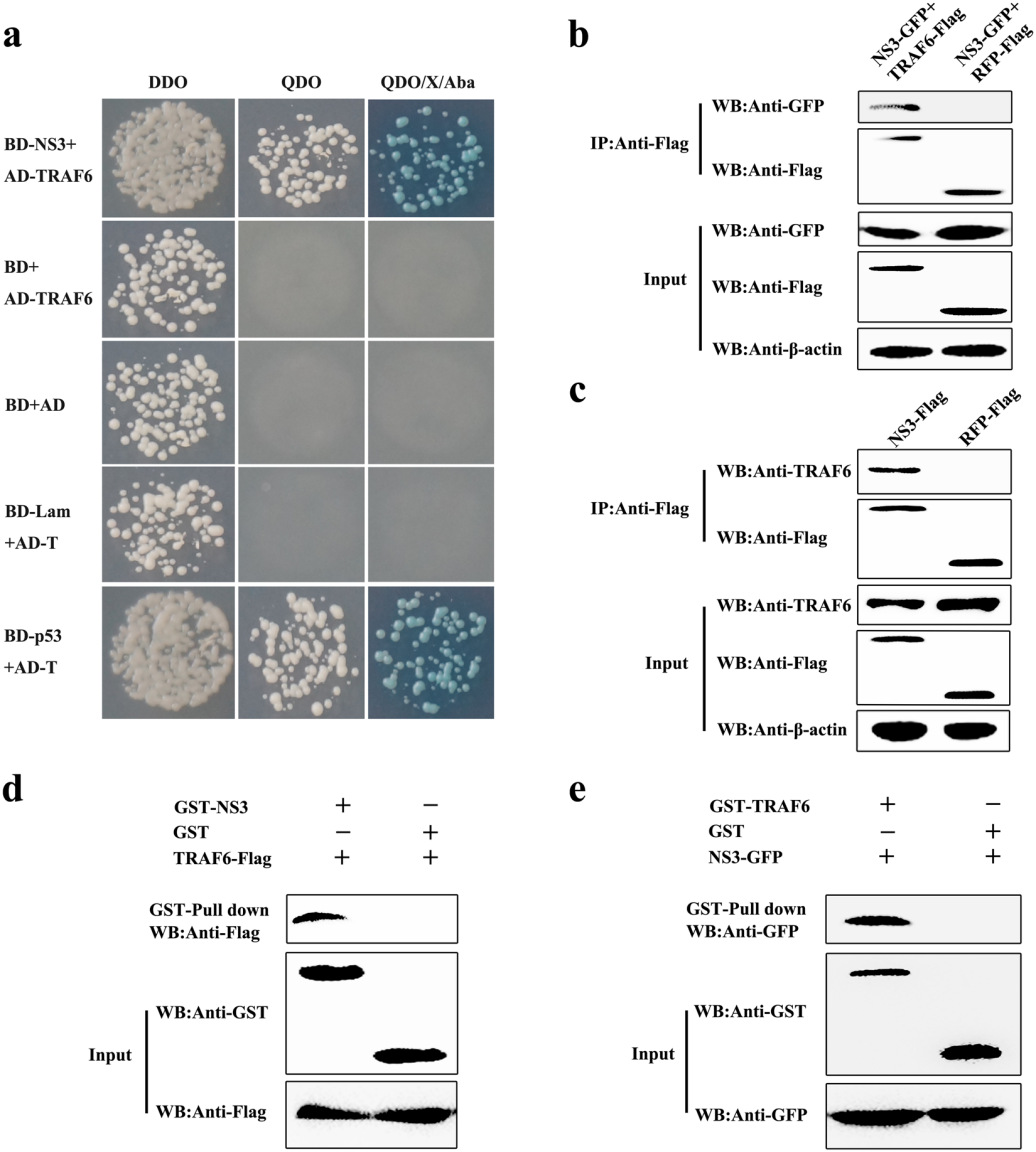
Interaction of NS3 with TRAF6. (**a**) Interaction of NS3 with TRAF6 in a yeast two-hybrid system. The yeast strain Y2HGold was cotransformed with the prey plasmid AD-TRAF6 and the bait plasmid BD or BD-NS3. Cotransformation with BD/AD, BD-Lamin/AD-T, and BD-p53/AD-T were used as a blank, negative and positive controls, respectively. (**b**) Exogenous co-IP analysis of NS3 and TRAF6 in PAMs. Cells were co-transfected with plasmids NS3-GFP and TRAF6-Flag. PAMs co-transfected with NS3-GFP and RFP-Flag were used as negative controls. A quarter of the cell extract was subjected to the input assay to assess β-actin, Flag-fusion, and GFP-fusion protein levels. The rest of the extract was subjected to IP assay. Western blot detected proteins with a mouse anti-GFP mAb and a rabbit anti-Flag pAb. (**c**) Endogenous co-IP analysis of NS3 and TRAF6 in PAMs. Cells were transfected with plasmid NS3-Flag, and RFP-Flag-transfected PAMs were used as negative controls. The input assay was performed using a quarter of the cell extract to assess β-actin, Flag fusion protein, and TRAF6 levels. The precipitated proteins were detected with a rabbit anti-TRAF6 pAb and a rabbit anti-Flag pAb. (**d**) GST-NS3 pull-down assay. The GST and GST-NS3 proteins expressed in *Escherichia coli* Rosetta (DE3) cells were immobilized on a glutathione agarose resin, followed by incubation of the resin with the cell lysates containing TRAF6-Flag protein. After washing, the bound proteins were detected by Western blot using a mouse anti-Flag mAb. The expression of input proteins (TRAF6-Flag, GST or GST-NS3) was confirmed by Western blot using a mouse anti-Flag mAb and a mouse anti-GST mAb, respectively. (**e**) GST-TRAF6 pull-down assay. The GST and GST-TRAF6 proteins expressed in *Escherichia coli* Rosetta (DE3) cells were immobilized on the glutathione-agarose resin. The resin conjugated with GST or GST-TRAF6 was incubated with the cell lysates containing NS3-GFP protein. After washing, the eluted proteins were detected by Western blot using a mouse anti-GFP mAb.

Yeast two-hybrid screening identified 26 proteins as having potential interactions with CSFV NS3 (Table 1). The identified proteins were predicted as being involved in DNA binding, RNA binding, metabolism, signalling pathways, ubiquitin-mediated proteolysis, and cancer-related pathways. Previously, our group focused on the TLR-mediated host innate immune response upon CSFV infection. CSFV Shimen infection results in a significant induction of TLR2, TLR4, and TLR7, but decreased of TLR3. Importantly, TLR3-mediated innate responses induced by poly(I:C) are inhibited in the Shimen infected porcine monocyte-derived macrophages (pMDMs). We also revealed that CSFV Shimen infection of pMDMs leads to the activation of MAPK signalling pathways, while it fails to activate NF-κB. Furthermore, the Shimen infection reduces interferon regulatory factor (IRF)3 expression, but enhances IRF7 expression, thereby affecting the production of type I IFN responses^21^. HCV infection suppresses host innate immune response by degrading TRAF6^23^. Among the identified proteins, we chose TRAF6 for further study due to its involvement in the NF-κB-signalling pathway and innate immune response. First, we verified interactions between TRAF6 and NS3 by the Y2H system. The yeast strain Y2HGold was co-transformed with the prey plasmid AD-TRAF6 and the bait plasmid BD-NS3 or BD. Co-transformations with BD-p53/AD-T, BD-Lam/AD-T, and BD/AD as positive, negative and blank controls, respectively, indicated that the experiments were successful (Fig. 1a).

**Table 1:** The results of the positive clones mating with NS3 BLAST to NCBI.

Protein No.	Protein name	Gene	NCBI protein accession no.		Max identity (%)	No. of clones
			Pig	Human		
1	ATPase, H+ transporting, lysosomal 21kDa, V0 subunit b	ATP6V0B	JAG69704	CAG33253.1	89	1
2	18S ribosomal RNA	RN18S	NR_046261.1	Not found	99	2
3	TNF receptor-associated factor 5	TRAF5	XP_003482803.1	NP_665702.1	92	2
4	eukaryotic translation elongation factor 1 epsilon 1	EEF1E1	NP_001230474.1	AAH05291.1	99	2
5	ribosomal protein L13	RPL13	NP_001230274.1	AAH93063.1	98	1
6	BMX non-receptor tyrosine kinase	BMX	XP_005673506.1	NP_975010.1	87	1
7	Mdm2, p53 E3 ubiquitin protein ligase homolog (mouse)	MDM2	NP_001098773.1	ACX31156.1	84	2
8	TNF receptor-associated factor 6	TRAF6	NP_001098756.1	NP_665802.1	90	2
9	ribosomal protein S3	RPS3	NP_001038066.1	AAH71917.1	99	1
10	seryl-tRNA synthetase	SARS	XP_001925705.3	AAI11399.1	100	1
11	ribosomal protein L10	RPL10	NP_001038008.1	CAG46866.1	99	1
12	annexin A8	ANXA8	NP_001230528.1	NP_001258631.1	96	1
13	proteasome (prosome, macropain) 26S subunit, non-ATPase, 11	PSMD11	XP_013845409.1	NP_001257411.1	97	1
14	eukaryotic translation initiation factor 3, subunit L	EIF3L	NP_001231015.1	NP_001229852.1	99	1
15	tyrosine3-monooxygenase/tryptophan 5-monooxygenase activation protein, beta polypeptide	YWHAB	JAG69041.1	AAH01359.1	99	1
16	cytochrome P450 1A1	CYP1A1	NP_999577.1	NP_000490.1	97	1
17	heat shock 70kDa protein 8	HSPA8	NP_001230836.1	AAH16660.1	99	1
18	NADH dehydrogenase (ubiquinone) 1 alpha subcomplex, 5	NDUFA5	XP_003134775.1	NP_004991.1	99	1
19	F-box and WD repeat domain containing 7, E3 ubiquitin protein ligase	FBXW7	XP_013834280.1	AAI43945.1	94	1
20	Ras association (RalGDS/AF-6) domain family member 3	RASSF3	XM_007115965.1	AAI00951.1	83	1
21	RAP1, GTP-GDP dissociation stimulator 1	RAP1GDS1	XP_013844849.1	AAH99845.	94	1
22	lectin, mannose-binding, 1	LMAN1	JAG69411.1	AAH17858.1	99	1
23	mucin 13, cell surface associated	MUC13	NP_001098763.1	AAQ89190.1	89	1
24	zinc finger protein 25	ZNF25	XP_005671049.1	AAH36038.1	86	2
25	TWIST neighbor	TWISTNB	NP_001230885.1	AAH14574.1	88	1
26	BCL2-like 13 (apoptosis facilitator)	BCL2L13	XP_003126643.3	XP_016884217.1	89	2

### NS3 binds to TRAF6 in cells

Co-immunoprecipitation (co-IP) assay was performed to validate potential interactions between NS3 and TRAF6. Expression of Flag fusion proteins, β-actin, NS3-GFP and TRAF6 was assessed using a quarter of the cells in each well of the 6-well plate in the input assay. For the exogenous co-IP assay, Western blot results showed that TRAF6-Flag coprecipitated NS3-GFP, but we observed no signal when using the RFP-Flag samples. Also, TRAF6-Flag and RFP-Flag were detected with a rabbit anti-Flag pAb due to the cross reaction of the 55 kDa heavy chain and 25 kDa light chain of lgG (Fig. 1b). For the endogenous co-IP assay, NS3-Flag and RFP-Flag were separately transfected into porcine alveolar macrophages (PAMs) to detect endogenous TRAF6, with results indicating that endogenous TRAF6 was co-immunoprecipitated with NS3-Flag but not RFP-Flag (Fig. 1c). These results revealed specificity in the interactions between NS3 and TRAF6 in cells.

### NS3 interacts with TRAF6 *in vitro*

For glutathione *S*-transferase (GST) pull-down experiments, generation of soluble GST-fusion proteins is crucial. Here, inducing expression of GST-NS3 occurs following the addition of 1 mM isopropyl β-D-1-thiogalactopyranoside (IPTG) and incubation for 6 h at 20 °C. Expressed GST-NS3 proteins were added to a Pierce spin column and were immobilized with glutathione agarose for 2 h, followed by addition of cell lysates containing TRAF6-Flag. Eluted proteins were analysed by Western blot using anti-Flag antibodies. We observed that TRAF6 was captured by NS3-GST rather than by GST alone (Fig. 1d). To confirm the interaction between NS3 and TRAF6, GST-TRAF6 proteins expressed in *Escherichia coli* Rosetta cells and the NS3-GFP protein expressed in HEK293T cells were subjected to the GST pull-down assay, with results showing that GST-TRAF6, not GST, interacted with NS3-GFP (Fig. 1e). These findings demonstrated that NS3 interacted with TRAF6 *in vitro*.

### TRAF6 overexpression inhibits CSFV replication

**Figure 2:**
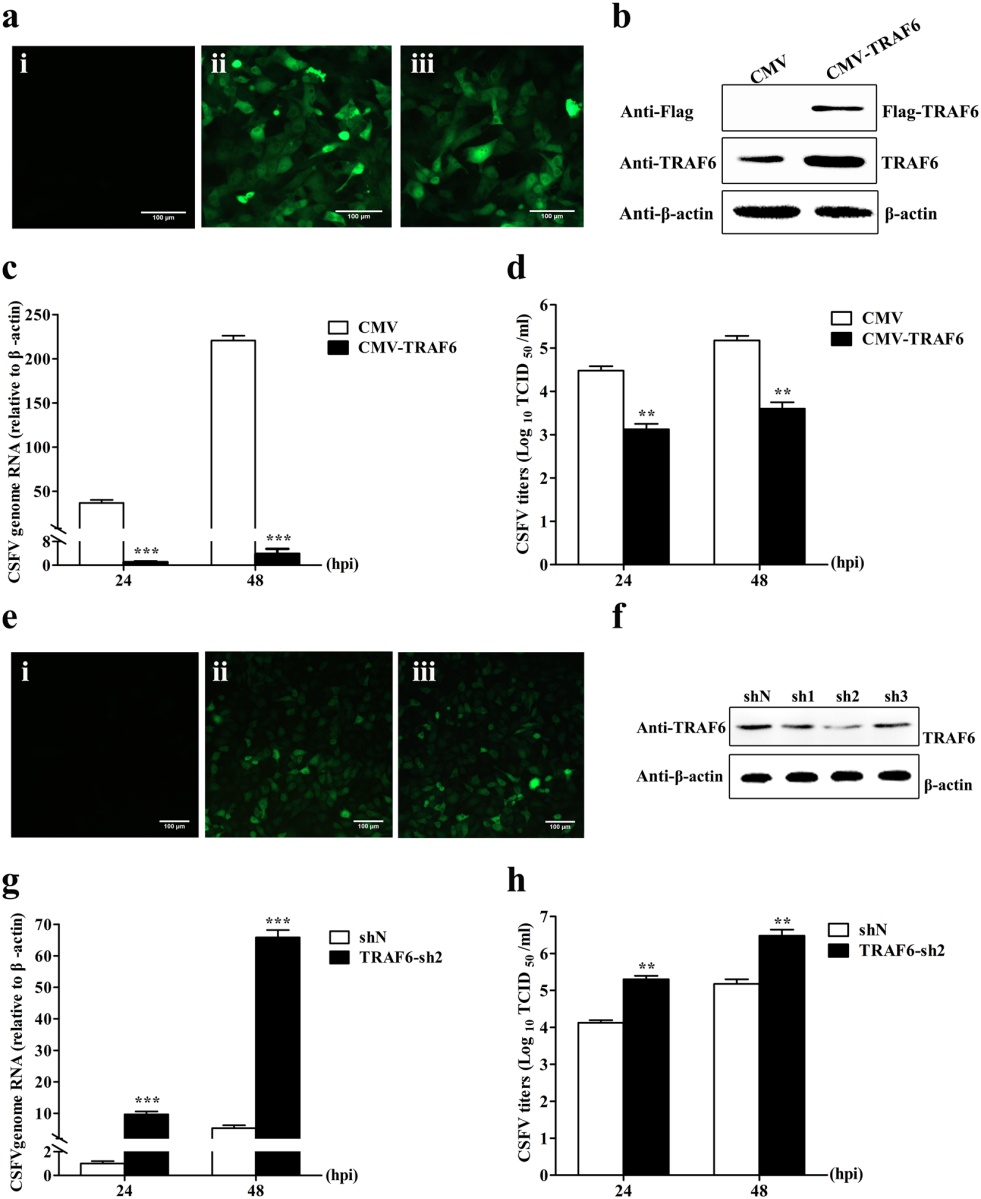
TRAF6 inhibits CSFV replication. PAMs stably overexpressing TRAF6 and knockdown of TRAF6 were constructed via lentivirus. (**a**) Confirmation of CMV-TRAF6 recombinant lentivirus infection by fluorescence detection of the GFP reporter expressed in PAMs. (i) Mock-infected PAMs. (ii) PAMs infected with lentiviruses expressing CMV. (iii) PAMs infected with lentiviruses expressing CMV-TRAF6. (**b**) Western blot for TRAF6 expression in stable CMV-TRAF6 cells. β-actin was used as an internal control. (**c**) CSFV genome RNA in TRAF6-overexpressing cells. Cells stably expressing CMV or CMV-TRAF6 were infected with CSFV. RT-PCR determined CSFV genome RNA levels at 24 and 48 hpi. (**d**) Infectious progeny viral titres in supernatants from TRAF6-overexpressing cells. Viral titres from the supernatant collected at 24 and 48 hpi were determined and expressed as TCID_50_/ml. (**e**) Confirmation of TRAF6-knockdown lentivirus infection by fluorescence detection of the GFP reporter expressed in PAMs. (i) Mock-infected PAMs. (ii) PAMs infected with lentiviruses expressing shN. (iii) PAMs infected with TRAF6-knockdown lentivirus. (**f**) Western blot for TRAF6 expression in TRAF6 knockdown cells. Cells stably expressing TRAF6-sh1, TRAF6-sh2, TRAF6-sh3, or shN were constructed, and Western blot determined endogenous TRAF6 expression. (**g**) CSFV genome RNA in TRAF6-sh2 cells. Stable shN and TRAF6-sh2 cells were infected with CSFV, and RT-PCR determined CSFV genome RNA levels at 24 and 48 hpi. (**h**) Viral titres of infectious progeny in supernatant from TRAF6-sh2 cells. Viral titres from the supernatant collected at 24 and 48 hpi were determined and expressed as TCID_50_/ml. Error bars represent the mean ± SD of three independent experiments. **P < 0.01 and ***P < 0.001 compared with the controls calculated using Student’s *t-test*.

To determine whether TRAF6 participates in CSFV replication, we constructed a stable cell line overexpressing TRAF6 (CMV-TRAF6 cells) via lentivirus, with PAMs stably transfected along with an empty vector CMV as a negative control (CMV cells). As shown in Fig. 2a, green fluorescence in CMV or CMV-TRAF6 cells was visible under an inverted fluorescence microscope (Nikon, Japan), whereas we detected no green fluorescence in mock-transfected PAMs (the rate of positively transfected cells was ~100%). Additionally, TRAF6 overexpression was confirmed by Western blot using a mouse anti-Flag mAb and a rabbit anti-TRAF6 pAb (Fig. 2b). We infected cells with CSFV at a multiplicity of infection of 0.1, and collected lysates and supernatant to detect CSFV genome RNA and viral titres, respectively. As shown in Fig. 2c, levels of CSFV genome RNA were 36.9 and 57.1 fold lower at 24 and 48 h postinfection (hpi), respectively, in CMV-TRAF6 cells as compared with levels observed in CMV cells. Additionally, viral titres of the infectious progeny in the culture supernatant of CMV-TRAF6 cells decreased by 22.5 and 37.5 fold at 24 and 48 hpi, respectively, as compared with levels observed in controls (Fig. 2d), indicating that TRAF6 overexpression reduced CSFV replication.

### TRAF6 knockdown promotes CSFV propagation

To explore how silencing TRAF6 expression influences CSFV replication, we constructed cell lines stably transfected with TRAF6 shRNAs (TRAF6-sh cells) and a negative control (shN cells). The rate of positively transfected cells was approximately 100% (Fig. 2e). The knockdown efficiency in TRAF6-sh2 cells was highest (65%), and these cells were thus used for subsequent experiments (Fig. 2f). Following CSFV infection, CSFV RNA levels in TRAF6-sh2 cells increased by 9.6 and 12.5 fold at 24 and 48 hpi, respectively, compared to levels observed in controls (Fig. 2g). Additionally, the viral titres of the infectious progeny in the culture supernatant of TRAF6-sh2 cells increased by 15.0 and 20.0 fold at 24 and 48 hpi, respectively, as compared with levels observed in controls (Fig. 2h), suggesting that TRAF6 inhibited CSFV replication and exhibited antiviral activity during CSFV infection.

### CSFV or NS3 degrades TRAF6

**Figure 3:**
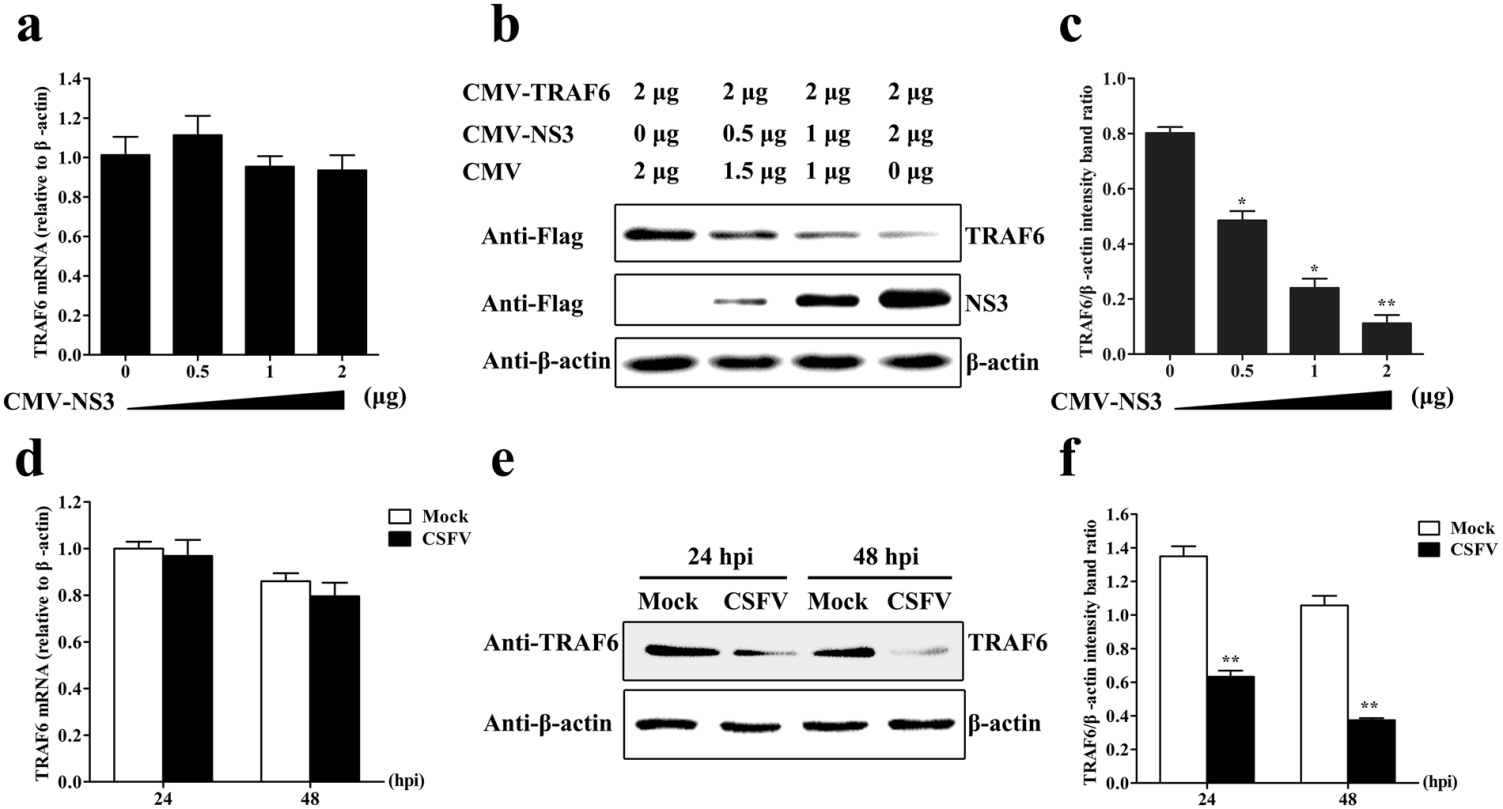
CSFV or NS3 degrades TRAF6. (**a**,**b**) TRAF6 mRNA and protein expression in NS3-expressing PAMs. PAMs were co-transfected with CMV-TRAF6 and a different amount of CMV-NS3. RT-PCR and Western blot analysed TRAF6 mRNA and protein expression at 24 hpt. β-actin served as an internal control. (**c**) The relative levels of TRAF6 protein were estimated by histograms representing density readings of the gel bands, and the ratios were calculated relative to β-actin control. (**d**,**e**) TRAF6 mRNA and protein expression in CSFV-infected PAMs. PAMs were mock-infected or CSFV-infected. RT-PCR and Western blot analysed TRAF6 mRNA and protein levels at 24 and 48 hpi. (**f**) The relative levels of TRAF6 protein were estimated by histograms representing density readings of the gel bands, and the ratios were calculated relative to β-actin control. The data represent the mean ± SD of three independent experiments. *P < 0.05 and **P < 0.01 calculated using Student’s *t-test*.

To investigate the effect of CSFV NS3 on TRAF6 protein levels, we co-transfected TRAF6-Flag with different amounts of CMV-NS3 into PAMs, and the cells were harvested to assess TRAF6 expression levels by RT-PCR and Western blot. Our results indicated no changes in TRAF6 mRNA levels, but lower TRAF6 protein levels coinciding with increases in NS3. The relative levels of TRAF6 protein were estimated by histograms representing density readings of the gel bands, and ratios were calculated relative to β-actin control (Fig. 3a–c). To determine whether TRAF6 expression levels are regulated by CSFV infection, mock- and CSFV-infected PAMs were harvested at the selected times to detect TRAF6 expression. We observed no obvious changes in TRAF6 mRNA levels following CSFV infection (Fig. 3d); however, TRAF6 protein levels were significantly lower in CSFV-infected cells than in mock-infected cells at 24 and 48 hpi (Fig. 3e and f). These findings revealed that CSFV or NS3 altered cellular TRAF6 protein levels without affecting mRNA expression, indicating a role in TRAF6 degradation. These results and our previous observations that TRAF6 inhibits CSFV replication suggested that TRAF6 and CSFV are co-antagonistic and that TRAF6 is important for CSFV evasion of host innate immunity for persistent infection.

### TRAF6 promotes NF-κB activation in PAMs

**Figure 4:**
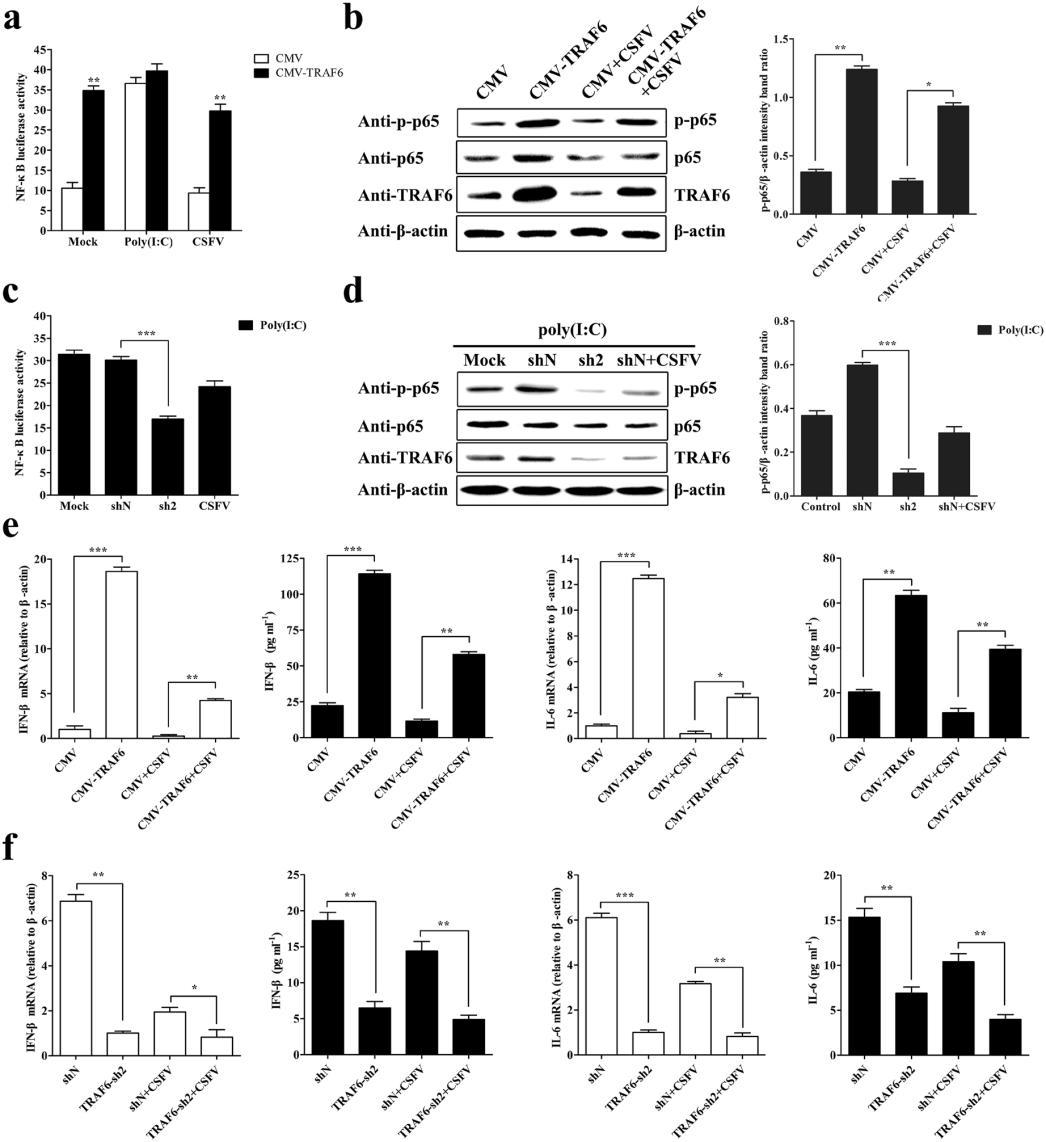
TRAF6 promotes NF-κB activation and increases IFN-β and IL-6 expression in PAMs. (**a**) TRAF6 overexpression promotes NF-κB activity. Stable cell lines were co-transfected with pNF-κB-luc and pRL-TK plasmids. At 12 hpt, the cells were mock-infected, CSFV-infected, or poly (I:C)-stimulated for an additional 12 h. The dual-luciferase reporter assay determined NF-κB luciferase activity. (**b**) Western blot for p65, p-p65, and TRAF6 expression in mock- and CSFV-infected CMV-TRAF6 cells. Cells stably expressing CMV or CMV-TRAF6 were mock- or CSFV-infected for 24 h, and the cell lysate was subjected to Western blot. (**c**) TRAF6 knockdown inhibits NF-κB activity initiated by poly (I:C). Stable shN and TRAF6-sh2 cells were treated with poly (I:C), with shN cells infected with CSFV for 24 h, and PAMs treated with poly (I:C) as a mock condition. Cell lysates were used to determine NF-κB luciferase activity. (**d**) Western blot for p65, p-p65, and TRAF6 expression in TRAF6-sh2 cells. Stable shN and TRAF6-sh2 cells were treated with poly (I:C), with shN cells infected with CSFV for 24 h, PAMs treated with poly (I:C) as a mock condition. Cell lysate was subjected to Western blot. The relative levels of p-p65 protein were estimated by histograms representing density readings of the gel bands, and the ratios were calculated relative to the β-actin control. (**e**) TRAF6 overexpression increases IFN-β and IL-6 expression in PAMs. Cells stably expressing CMV or CMV-TRAF6 were mock- or CSFV-infected for 24 h, followed by extraction of total RNA to determine relative IFN-β and IL-6 mRNA expression. The cell culture supernatant was used to determine IFN-β and IL-6 protein levels. (**f**) TRAF6 knockdown decreases IFN-β and IL-6 expression in PAMs. The stable shN and TRAF6-sh2 cells were mock- or CSFV-infected for 24 h. Relative IFN-β and IL-6 mRNA expression and protein levels were determined as described. The data represent the mean ± SD of three independent experiments. *P < 0.05, **P < 0.01 and ***P < 0.001 compared with the controls calculated using Student’s *t-test*.

Having validated TRAF6 degradation by CSFV and inhibition of CSFV propagation by TRAF6, we investigated TRAF6-mediated antiviral response in cells by detecting NF-κB activity and the expression of its downstream targets IFN-β and IL-6. To determine NF-κB activity, an NF-κB-luciferase (NF-κB-luc) assay was performed using stable CMV-TRAF6 cells, with NF-κB activity subsequently evaluated as 3.3 fold higher than levels observed in controls. Additionally, stable CMV-TRAF6 cells infected with CSFV Shimen strain showed 3.1 fold higher NF-κB activity than that observed in controls (Fig. 4a). In stable knockdown TRAF6-sh2 cells treated with poly (I:C) (an agonist of NF-κB activation), we observed a 1.8 fold decrease in NF-κB activity as compared with levels in shN cells treated with poly (I:C) (Fig. 4c). Additionally, we determined TRAF6, NF-κB p65 and phosphorylated p65 (p-p65) levels in stable CMV-TRAF6 and TRAF6-sh2 cells, revealing higher levels of p-p65 in CMV-TRAF6 cells and decreased p-p65 levels in TRAF6-sh2 cells than those observed in controls (Fig. 4b and d), indicating that TRAF6 promoted NF-κB activation.

We then determined alterations in the levels of the downstream NF-κB targets IFN-β and IL-6 by RT-PCR and ELISA. In stable CMV-TRAF6 cells, IFN-β mRNA and protein levels were 18.6 and 5.2 fold higher, and IL-6 mRNA and protein levels were 12.5 and 3.1 fold higher, respectively, than levels observed in control cells (Fig. 4e). By contrast, in stable TRAF6-sh2 cells, IFN-β mRNA and protein levels were 6.9 and 2.9 fold lower, and IL-6 mRNA and protein levels were 6.1 and 2.2 fold lower, respectively, than the levels observed in controls (Fig. 4f).

### NF-κB activation inhibits CSFV replication in PAMs

**Figure 5:**
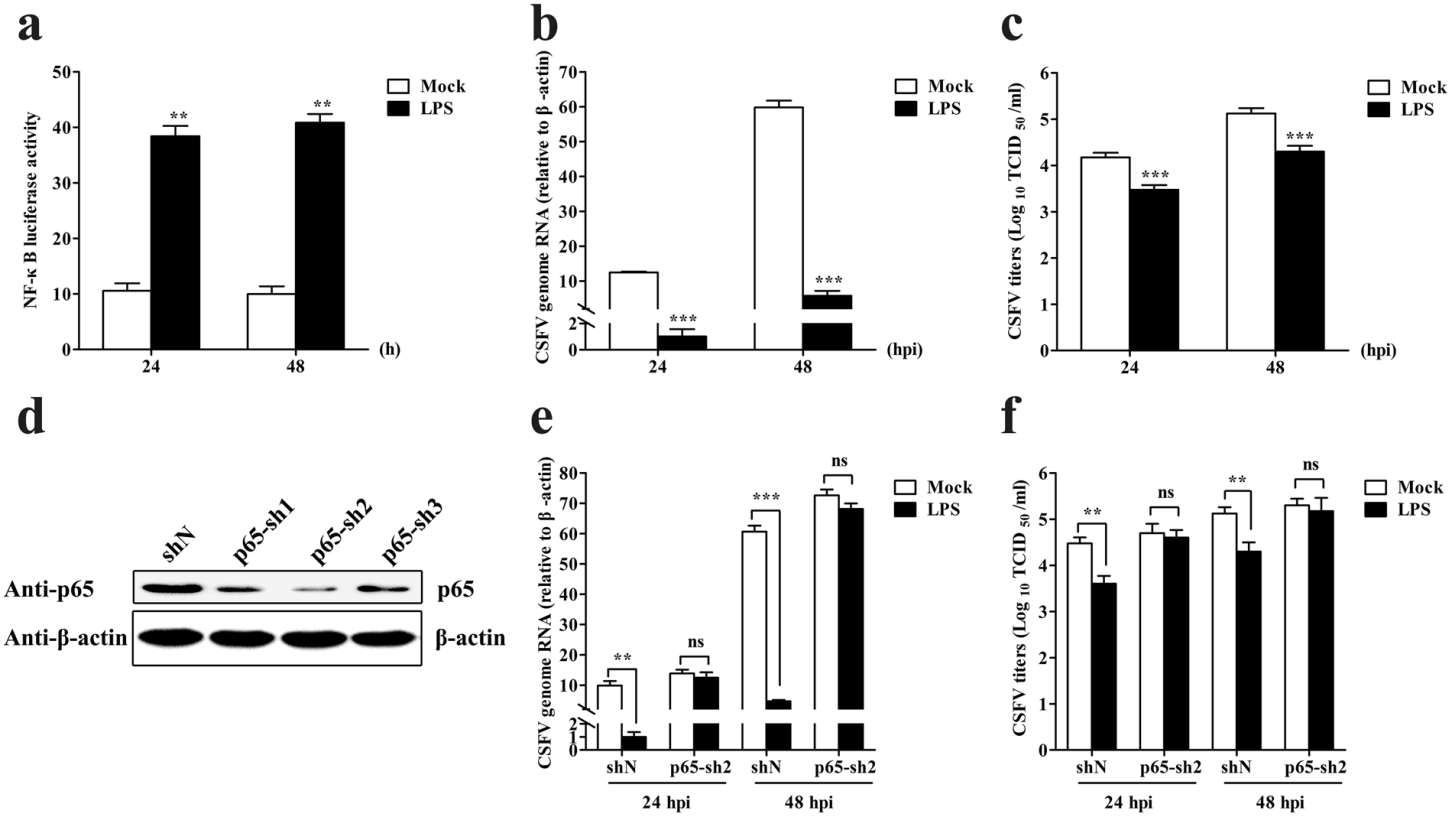
LPS inhibits CSFV replication in PAMs via the NF-κB-signalling pathway. (**a**) LPS treatment increases NF-κB activity in PAMs. PAMs were treated with LPS, and cell lysates were subjected to dual-luciferase reporter assays to determine NF-κB luciferase activity at 24 h and 48 h. (**b**,**c**) LPS treatment decreases CSFV replication in PAMs. RT-PCR analysed CSFV genome RNA, and viral titres in the supernatant were assessed and expressed as TCID_50_/ml in LPS-treated PAMs at 24 and 48 hpi. (**d**) p65 knockdown by shRNA in PAMs. PAMs were transfected with p65-sh1, p65-sh2, p65-sh3 or shN and the cell lysates were subjected to determination of endogenous p65 expression by Western blot at 48 hpt. (**e**,**f**) LPS does not inhibit CSFV replication in p65-knockdown PAMs. PAMs treated with LPS were transfected with p65-sh2 and infected with CSFV. CSFV genome RNA and viral titres were determined at 24 and 48 hpi. The data represent the mean ± SD of three independent experiments. **P < 0.01 and ***P < 0.001 calculated using Student’s *t-test*. ns: not significant.

To investigate the relationship between NF-κB activation and CSFV replication, we examined CSFV genome RNA, and viral titres in CSFV-infected PAMs treated with the NF-κB agonist lipopolysaccharide (LPS) at 24 and 48 hpi, with DMSO treatment applied as a mock condition. NF-κB activity was elevated in LPS-treated PAMs (Fig. 5a), indicating activation of NF-κB by LPS. Additionally, viral genome RNA and viral titres were lower in LPS-treated PAMs as compared with levels observed in controls (Fig. 5b and c), indicating that NF-κB activation inhibited CSFV replication in PAMs.

Furthermore, to investigate whether LPS inhibits CSFV replication via the NF-κB-signalling pathway, we knocked down p65 using shRNA and treated the PAMs with LPS to determine CSFV replication. As shown in Fig. 5d, p65-sh2-knockdown efficiency was highest (72%), resulting in use of this shRNA for subsequent experiments. Interestingly, we observed that LPS treatment did not reduce the amount of viral genome RNA and viral titres in p65-sh2-transfected PAMs (Fig. 5e and f), suggesting that LPS inhibited CSFV replication via the NF-κB-signalling pathway in PAMs.

### TRAF6 does not inhibit CSFV replication following inhibition of NF-κB activation

**Figure 6:**
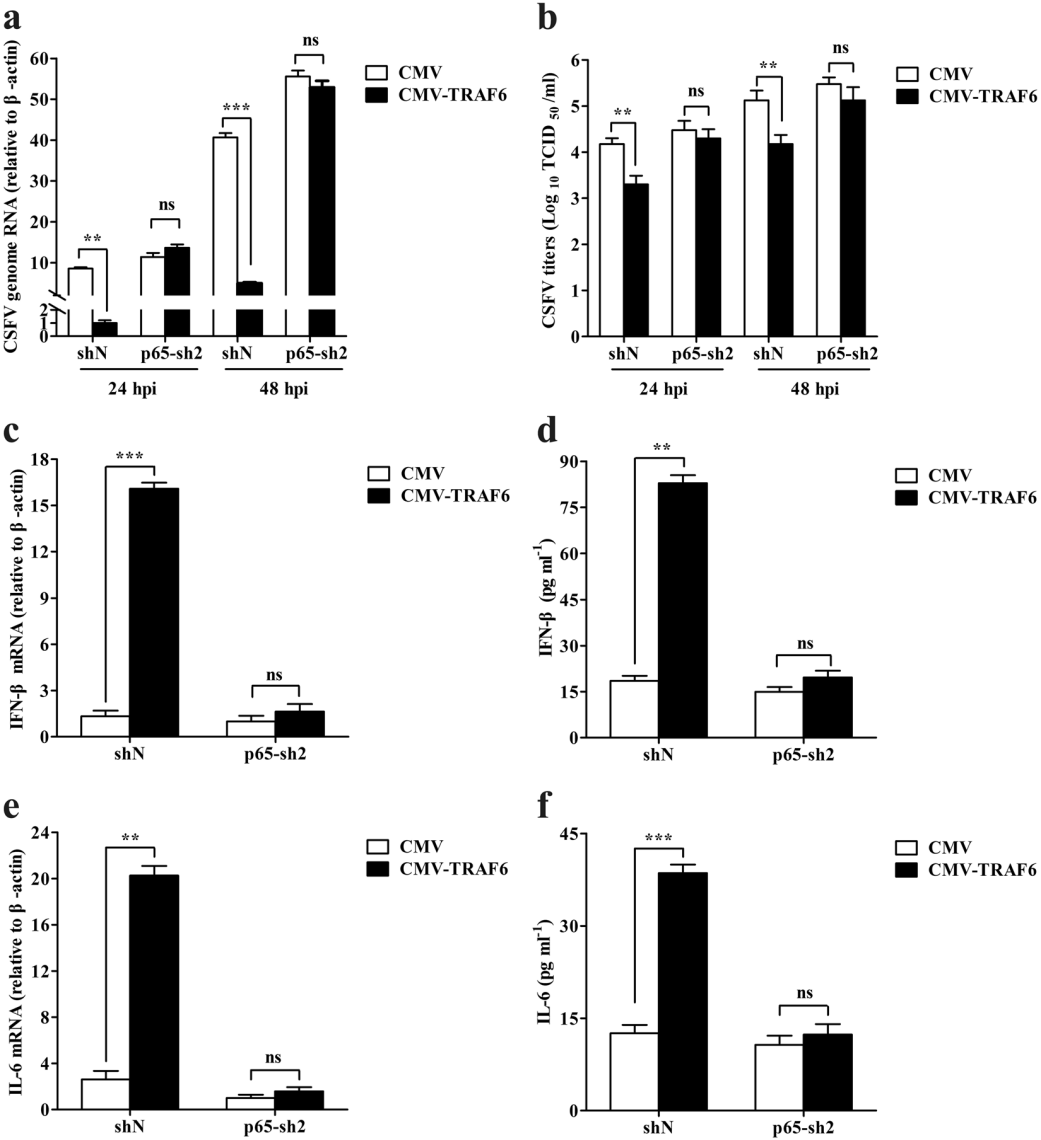
TRAF6 overexpression does not inhibit CSFV replication following inhibition of NF-κB activation in PAMs. (**a**,**b**) CSFV replication in p65-knockdown CMV-TRAF6 cells. Cells stably expressing CMV or CMV-TRAF6 were transfected with p65-sh2 or shN, followed by CSFV infection. RT-PCR analysed CSFV genome RNA, and viral titres in the supernatant were assessed and expressed as TCID_50_/ml at 24 and 48 hpi. (**c**–**f**) Expression of IFN-β and IL-6 in p65-knockdown CMV-TRAF6 cells. Cells stably expressing CMV or CMV-TRAF6 were transfected with p65-sh2 or shN. Cell lysates and culture supernatant were subjected to RT-PCR and ELISA to determine IFN-β and IL-6 mRNA and protein levels at 24 h, respectively. The data represent the mean ± SD of three independent experiments. **P < 0.01 and ***P < 0.001 calculated using Student’s *t-test*. ns: not significant.

TRAF6 inhibits CSFV replication and promotes NF-κB activation. Therefore, we investigated whether TRAF6 inhibits CSFV replication via NF-κB activation. Cells stably expressing CMV or CMV-TRAF6 were subjected to p65 knockdown with p65-sh2, followed by CSFV infection. Interestingly, we observed that TRAF6 did not reduce CSFV genome RNA or viral titres in p65-sh2-transfected CMV-TRAF6 cells at 24 and 48 hpi, whereas TRAF6 reduced levels of CSFV genome RNA and viral titres in shN-transfected CMV-TRAF6 cells (Fig. 6a and b). Additionally, the mRNA and protein levels of IFN-β and IL-6 were elevated in shN-transfected CMV-TRAF6 cells, but were unaltered in p65-sh2-transfected CMV-TRAF6 cells (Fig. 6c–f). These results demonstrated that TRAF6 was unable to suppress CSFV replication after blockade of the NF-κB-signalling pathway. These data confirmed that TRAF6 inhibited CSFV replication via the NF-κB-signalling pathway, accompanied by IFN-β and IL-6 expression.

## Discussion

CSFV is capable of establishing persistent infection through attenuating the immune response and prevention of host cell apoptosis^24, 25^. CSFV E^rns^ suppresses the expression of IFN-β and cell apoptosis^25^, and CSFV N^pro^ limits type I IFN induction through proteasomal degradation of IRF3 and interaction with IRF7^26, 27^. CSFV NS3 is a multifunctional, non-structural protein that plays a major role in CSFV replication^3, 4, 7^. In HCV, NS3 modulates cell signalling and host-cell responses through its interaction with various cellular proteins, including binding to host apolipoprotein B mRNA-editing enzyme catalytic subunit 3G protein to inhibit HCV replication^28, 29^. However, whether CSFV NS3 attenuates the immune response remains unclear. Additionally, no studies are investigating host-cell protein interactions with CSFV NS3. Here we verified 26 host-cell proteins as interacting with CSFV NS3 according to yeast two-hybrid results, with the identified proteins predicted to be involved in metabolism, endocytosis, apoptosis, heat-shock response, and immune response according to bioinformatics analysis. Furthermore, TRAF6 was verified as interacting with CSFV NS3 by co-IP and GST pull-down assays. These results provide the empirical foundation for further research on the function of CSFV NS3, and it is the first report that TRAF6 interacts with CSFV NS3. Moreover, we showed that TRAF6 inhibited CSFV replication and that both CSFV and NS3 alone were capable of degrading TRAF6 in PAMs. Additionally, CSFV NS3 is dose-dependent for degradation of TRAF6. However, reduced TRAF6 levels resulted in downregulation of IFN-β and IL-6 expression, which contributed to CSFV persistent replication. These results offered insight into the mechanisms related to CSFV evasion of host antiviral responses via TRAF6 degradation by CSFV NS3.

TRAF6 both mediates signalling associated with the TNF-receptor superfamily and participates in signal transduction linked to the IL-1R/TLR superfamily^30, 31^. It was previously shown that downregulation of TRAF6 decreases cell viability, suppresses cell proliferation, and promotes cell apoptosis in lung cancer cells^32^. Furthermore, TRAF6 mediates TLR-signalling pathways and NF-κB activation to modulate the expression of antiviral cytokines^33, 34^. Recent studies demonstrated that TRAF6 expression is reduced in chronic HCV patients, and TRAF6 knockdown results in increased HCV RNA levels in myeloid dendritic cells^35, 36^. In this study, cellular protein TRAF6 interacted with the CSFV NS3 and inhibited CSFV replication. Considering this with the results, the participation of TRAF6 in CSFV life cycle indicates that this protein plays a potential role in host immune defence and anti-CSFV strategies.

Previous studies showed that TRAF6 inhibits CSFV replication; however, the precise mechanism required further investigation. TRAF6 downregulation of decreases levels of p-p65 in the lung cancer cell line A549. TRAF6 overexpression is associated with lung cancer tumourigenesis and invasion^32^. Additionally, TRAF6 is a multifunctional adaptor protein involved in the NF-κB-signalling pathway^37^. NF-κB is a ubiquitous transcription factor that plays a central role in immunity. Viruses elicit the expression and release of a range of cytokines via the NF-κB-signalling pathway as part of the primary host defense mechanism. A previous study reported that CSFV failed to activate the NF-κB-signalling pathway^20, 21^. Our findings showed that TRAF6 overexpression promoted NF-κB activation, and resulted in upregulation of IFN-β and IL-6 expression in PAMs. Moreover, TRAF6 knockdown decreased NF-κB activation induced by poly (I:C) and downregulated IFN-β and IL-6 expression. IFN-β, an antiviral cytokine, and IL-6, involved in immune responses, might contribute to the suppression of CSFV replication. The results implied that TRAF6 degradation might play a major role in inhibiting NF-κB-mediated signalling during CSFV infection. These findings agreed with observations that some RNA viruses, such as Sendai virus or vesicular stomatitis virus could induce proteasome-dependent or -independent TRAF6 degradation to impair NF-κB activation and production of type I IFNs and proinflammatory cytokines^38^.

Activation of NF-κB signalling via TNF-α inhibits CSFV replication in PK-15 cells^22^. Here, our results showed that activation of NF-κB signalling via LPS inhibited CSFV replication in PAMs, whereas LPS failed to inhibit CSFV replication following inhibition of NF-κB activation by p65 knockdown, indicating that CSFV was sensitive to NF-κB activation in PAMs, and that LPS treatment inhibited CSFV replication via activation of NF-κB signalling. Furthermore, we revealed that TRAF6 was unable to inhibit CSFV replication and increase IFN-β and IL-6 expression following inhibition of NF-κB activation. These findings suggested a possible mechanism involving TRAF6 activation of NF-κB signalling to increase production of IFN-β and IL-6 for inhibiting CSFV replication. It has reported that HCV suppresses host innate immune response via induction of autophagic degradation of TRAF6^23^. Further investigation is required to determine the TRAF6-degradation pathway, whether CSFV NS3 interacts with other TRAFs, and the roles of TRAF6 in adaptive immunity^39^.

In conclusion, our findings demonstrated that TRAF6 interacted with CSFV NS3 to inhibit CSFV replication via the NF-κB-signalling pathway. These results provided insight into the mechanisms associated with CSFV evasion of innate immune responses and continuous infection, as well as potential antiviral strategies focused on attenuating CSFV replication.

## Materials and Methods

### Cells and virus

PAM cells (ATCC; CRL-2845) were cultured in RPMI 1640 medium (Gibco, UK). Swine testicular (ST) cells and human embryonic kidney (HEK293T) cells were grown in Dulbecco’s minimal essential medium (DMEM) (Gibco, UK) with 10% fetal bovine serum (FBS) (Biowest, France). CSFV (Shimen strain) purchased from the Control Institute of Veterinary Bio-products and Pharmaceuticals (China). All the experiments related to CSFV were performed in the P3 biosafety laboratory abiding strictly by Laboratory Biosafety Manual in our lab.

### Plasmid construction

CSFV NS3 was amplified by polymerase chain reaction (PCR) and cloned into pGBKT7 (BD), pEGFP-N1, the lentivector pCDH-CMV-MCS-EF1 with a Flag-tag and pGEX-6P-1 to generate pGBKT7-NS3 (BD-NS3), NS3-GFP, CMV-NS3, and GST-NS3, respectively. TRAF6 was amplified by PCR from PAMs cDNA and cloned into pCDH-CMV-MCS-EF1 with a Flag-tag and pGEX-6P-1 to generate CMV-TRAF6 and GST-TRAF6. RFP was amplified and was cloned into pCDH-CMV-MCS-EF1 with a Flag-tag to generate RFP-Flag. Three pairs of shRNAs targeting TRAF6 or p65 and a negative control shN were predicted (http://rnaidesigner.thermofisher.com/) and designed. After annealing, the fragments were cloned into pCDH-U6-MCS-EF1-GreenPuro to create TRAF6-sh1, TRAF6-sh2, TRAF6-sh3, p65-sh1, p65-sh2, p65-sh3 and shN lentivectors, respectively. All primers are listed in Table 2, and restriction digestion and sequencing confirmed all plasmids.

**Table 2:** Primers used in this study.

primers	Sequence (5′ → 3′)	Purpose
NS3-GFP-F	CATGCTAGCGATGGGGCCTGCCGTTTGCAAGAAG	Amplification of *NS3*
NS3-GFP-R	AACTGCAGTAGACCAACTACTTGTTTTAGTGCTCTGCC	
BD-NS3-F	GGAATTCCATATGCCTAAGAAAAAGCGCAAAGTTGGGCCTGCCGTTTGCAAGAAG	Amplification of *NS3*
BD-NS3-R	AACTGCAGTAGCTCCTTCAATTCTGTCTCCTTCCCCTC	
GST-NS3-F	ACGCGTCGACTCGGGCCTGCCGTTTGCA	Amplification of *NS3*
GST-NS3-R	ATAAGAATGCGGCCGCTCATAGACCAACTACTTGTTTTAGTGC	
CMV-NS3-F	CTAGCTAGCATGGGGCCTGCCGTTTGC	Amplification of *NS3*
CMV-NS3-R	ATAAGAATGCGGCCGCTTACTTATCGTCGTCATCCTTGTAATCTAGACCAACTACTTG	
CMV-TRAF6-F	GCTCTAGAATGAGTCTGCTACATTGTGAAAA	Amplification of *TRAF6*
CMV-TRAF6-R	GGAATTCTTACTTATCGTCGTCATCCTTGTAATCTGTCCCCGAGTCTGTACTTC	
**GST-TRAF6-F**	GGAATTCATGAGTCTGCTACATTGTGAAAA	Amplification of *TRAF6*
**GST-TRAF6-R**	CCGCTCGAGCTATGTCCCCGAGTCTGTACTTC	
β-actin-F	CAAGGACCTCTACGCCAACAC	Quantitative real-time PCR for detection of β-actin
β-actin-R	TGGAGGCGCGATGATCTT	
CSFV-F	GATCCTCATACTGCCCACTTAC	Quantitative real-time PCR for detection of CSFV
CSFV-R	GTATACCCCTTCACCAGCTTG	
IFN-β-F	CATCCTCCAAATCGCTCTCC	Quantitative real-time PCR for detection of IFN-β
IFN-β-R	CTGACATGCCAAATTGCTGC	
IL-6-F	GAGCCCACCAGGAACGAAAGAG	Quantitative real-time PCR for detection of IL-6
IL-6-R	GCAGTAGCCATCACCAGAAGCA	
**TRAF6-sh1-F**	GATCCGCCTGCATCATCAAGTCAATATCAAGAGTATTGACTTGATGATGCAGGCTTTTTG	Knockdown of TRAF6
**TRAF6-sh1-R**	AATTCAAAAAGCCTGCATCATCAAGTCAATACTCTTGATATTGACTTGATGATGCAGGCG	
**TRAF6**-sh2-F	GATCCGCGTGATATGCTTCCCTATGATCAAGAGTCATAGGGAAGCATATCACGCTTTTTG	Knockdown of TRAF6
**TRAF6**-sh2-R	AATTCAAAAAGCGTGATATGCTTCCCTATGACTCTTGATCATAGGGAAGCATATCACGCG	
**TRAF6**-sh3-F	GATCCGCGCTGTGCCAACTACATATCTCAAGAGGATATGTAGTTGGCACAGCGCTTTTTG	Knockdown of TRAF6
**TRAF6**-sh3-R	AATTCAAAAAGCGCTGTGCCAACTACATATCCTCTTGAGATATGTAGTTGGCACAGCGCG	
p65-sh1-F	GATCCGAAAGACTGCCGGGATGGCTTCTATCAAGAGATAGAAGCCATCCCGGCAGTCTTTCTTTTTG	Knockdown of p65
p65-sh1-R	AATTCAAAAAGAAAGACTGCCGGGATGGCTTCTATCTCTTGATAGAAGCCATCCCGGCAGTCTTTCG	
p65-sh2-F	GATCCGCGAGCCCATGGAATTCCAGTACTTCAAGAGAAGTACTGGAATTCCATGGGCTCGCTTTTTG	Knockdown of p65
p65-sh2-R	AATTCAAAAAGCGAGCCCATGGAATTCCAGTACTTCTCTTGAAGTACTGGAATTCCATGGGCTCGCG	
p65-sh3-F	GATCCGCATCATGAAGAAGAGTCCTTTCAACAAGAGTTGAAAGGACTCTTCTTCATGATGCTTTTTG	Knockdown of p65
p65-sh3-R	AATTCAAAAAGCATCATGAAGAAGAGTCCTTTCAACTCTTGTTGAAAGGACTCTTCTTCATGATGCG	

### Real-time (RT)-PCR

The relative mRNA expression of CSFV, TRAF6, IFN-β and IL-6 were tested by RT-PCR using specific primers (Table 2). The cells were lysed using TRIzol for total cellular RNA. Subsequently, the cDNA was synthesized by reverse transcription using the PrimeScript RT reagent kit (Vazyme, China). RT-PCR was performed with GoTaq® Master Mix (Promega, USA) according to the manufacturer’s protocol. β-actin, a housekeeping gene, served as the internal control to normalize the relative expression of each gene. Relative transcript levels were analysed using the ΔΔCt method as specified by the manufacturer^40^.

### Western blot and enzyme-linked immunosorbent assay (ELISA)

Cell lysates were prepared in radioimmunoprecipitation (RIPA) buffer with protease inhibitor phenylmethanesulfonyl fluoride (PMSF) and Halt™ phosphatase inhibitor cocktail (Roche, Switzerland). The protein concentration was determined with BCA Protein Assay Kit. The samples were separated by 10% SDS-PAGE, followed by transfer onto polyvinylidene difluoride (PVDF) membranes. After blocking with 5% skim milk at room temperature for 2 h, the membranes were incubated with primary antibodies overnight at 4 °C. Primary antibodies used include rabbit anti-TRAF6 polyclonal antibody (pAb), rabbit anti-NF-κB p65 pAb, rabbit anti-phospho-NF-κB p65 pAb, rabbit anti-Flag pAb (SIGMA, USA), mouse anti-GST monoclonal antibody (mAb), mouse anti-GFP mAb and mouse anti-Flag mAb (CWBIO, China). After five washes with TBST, the membranes were incubated with horseradish peroxidase (HRP)-conjugated goat anti-rabbit (or mouse) IgG (Vazyme, China) secondary antibody for 2 h at room temperature. After another five washes, the signal was detected using an image analysis system (Bio-Rad, USA). The secreted protein levels of IFN-β and IL-6 in cell culture supernatant were determined with IFN-β antibody (LSBio) and IL-6 antibody (R & D Systems), respectively. All samples were in triplicate and read at 450 nm using a Multiskan FC Microplate Photometer (Thermo, USA).

### Indirect immunofluorescence assay (IFA)

IFA determined CSFV titres in the culture supernatant. ST cells were infected with the infectious progeny viruses collected from the culture supernatant. At 48-hour post infection (hpi), cells were fixed with 1:1 stationary liquid (methanol: acetone) at 4 °C for 20 min. Following three washes with PBS, the fixed cells were permeated with 1% Triton X-100 at 4 °C for 20 min. After three washes, the cells were incubated with 5% skim milk powder at 37 °C for 2 h, followed by incubation with positive CSFV serum at 4 °C overnight. After three washes, the cells were incubated with rabbit anti-pig IgG (whole molecule)-FITC antibody (Sigma, USA) at 37 °C for 2 h. Immunofluorescence was observed using a fluorescence microscope (Nikon, Japan). Mock-infected cells were used as controls to establish background staining levels.

### Yeast two-hybrid screening

For the yeast two-hybrid assay, a Matchmaker Gold Yeast Two-Hybrid system (Clontech, USA) was applied to screen the host proteins interacting with NS3. Briefly, the yeast strain Y2HGold transformed with the bait plasmid pGBKT7-NS3 was mated with Y187 containing a PAM cDNA library cloned into the pGADT7-Rec (AD) vector according to the manufacturer’s instructions. Heterozygotes were plated on double-dropout (DDO) plates lacking Leu and Trp. The clones were then transferred to quadruple-dropout plates lacking His, Leu, Trp, and Ade containing X-alpha-galactosidase (X-α-Gal) and aureobasidin A (QDO/X-α-Gal/Aba) three times. The yeast plasmids were extracted, and the prey plasmids were isolated with ampicillin by transformation into *Escherichia coli* DH5α. Sequencing identified the prey plasmids, and the sequences were aligned using the Basic Local Alignment Search Tool (BLAST) on the NCBI website against the human non-RefSeq and pig non-RefSeq databases. To confirm the interaction between NS3 and the cellular proteins, Y2HGold was cotransformed with the bait and prey plasmids. The transformants were grown on DDO and QDO/X-α-Gal/Aba plates. Cotransformation with BD-p53/AD-T (simian virus 40 [SV40] large T antigen), BD-Lam (human lamin C protein)/AD-T, and BD/AD served as positive, negative and blank controls, respectively.

### Co-immunoprecipitation assays

For co-immunoprecipitation (co-IP) assays, endogenous verification was performed in conjunction with exogenous expression. For exogenous verification, PAMs (10^5^ cells) were cotransfected with 2 μg of NS3-EGFP and 2 μg of CMV-TRAF6 plasmids. After 36 h, the cells were washed with PBS and were harvested with Western blot and IP lysis buffer (Beyotime, China) containing PMSF. After centrifugation for 30 min at 4 °C, a quarter of the supernatant was subjected to input assays. The rest were used for co-IP experiments with ANTI-FLAG M2 Affinity Gel (Sigma, USA) according to the manufacturer’s instructions. In brief, 50 μL of resin stored in 50% glycerol was centrifuged for 30 s at 9000 × *g* and rinsed twice with 1 mL of TBS. The cell lysate was added to the equilibrated resin and rocked gently overnight at 4 °C. The resin was washed three times with 1 mL of TBS and resuspended with 2 × SDS sample buffer for Western blot with anti-GFP and anti-Flag antibodies. For further endogenous verification, PAMs (10^5^ cells) were transfected with 4 μg of CMV-NS3 plasmid, and the samples were detected with rabbit anti-TRAF6 and anti-Flag pAb. The other steps were similar to those of exogenous verification.

### GST pull-down experiment

GST-NS3 protein was expressed in *Escherichia coli* Rosetta and TRAF6-Flag protein was expressed in HEK293T cells for the GST pull-down experiment. In the prokaryotic expression system, the induction temperature, induction time, IPTG concentration and expressing strains were evaluated for soluble GST-fusion protein. The Pierce GST Protein Interaction Pull-Down Kit (Thermo, USA) was used according to the manufacturer’s instructions. Briefly, GST or GST-NS3 protein expressed in *Escherichia coli* was treated with pull-down lysis buffer and then immobilized on equilibrated glutathione agarose resin for 2 h at 4 °C. The beads were washed five times with 1:1 wash solution (TBS: Pull-Down Lysis Buffer). Then, HEK293T cell lysates containing TRAF6-Flag were added and were incubated overnight at 4 °C. Protein samples were washed five times followed by elution with Glutathione Elution Buffer. Western blot detected the eluted proteins. GST-TRAF6 expressed in *Escherichia coli* Rosetta and NS3-GFP expressed in HEK293T cells were also used to further verify the interaction between NS3 and TRAF6 in the GST pull-down assay.

### Construction of stable cell lines exhibiting TRAF6 overexpression or knockdown

HEK293T cells were co-transfected with 2 μg CMV-TRAF6 or TRAF6 shRNA along with 0.67 μg pGag/Pol, 0.67 μg pRev, and 0.67 μg pVSV-G plasmids. At 16 h post-transfection (hpt), the medium was replaced with advanced DMEM supplemented with 2% FBS, 0.01 mM L-α-phosphatidylcholine, 0.01 mM cholesterol (Sigma, USA), 4.0 mM L-glutamine and 1:1000 diluted chemically defined lipid (Invitrogen, USA). After 48 h of incubation, the culture supernatant was centrifuged and was collected as the lentivirus. Lentiviral titres were determined by tissue culture infectious dose (TCID_50_) in HEK293T cells. PAMs in 6-well plates were transduced with the lentiviruses at 10 transduction units per cell, and polybrene was added to enhance infection rate. After 8–10 h, the medium was replaced with fresh medium and incubation for an additional 48 h. Positive cells were selected with puromycin (Thermo, USA) at 6 μg/ml final concentration to generate the stable cell lines for TRAF6 overexpression (CMV-TRAF6 cells) or knockdown (TRAF6-sh2 cells). The empty vector pCDH-CMV-MCS-EF1-GreenPuro (CMV) and random sequence vector (shN) was treated equally as controls. The cell lines were used to examine TRAF6 expression and for further experiments.

### NF-κB reporter assay

Stable cell lines (CMV-TRAF6 cells or TRAF6-sh2 cells) in 24-well plates were transfected with 500 ng pNF-κB-luc and 50 ng pRL-TK Renilla luciferase (Promega, USA), the pRL-TK plasmid as a control for transfection efficiency. At 12 hpt, cells were mock-infected, CSFV-infected, or ligand-stimulated for an additional 12 h. The activities of Firefly and Renilla luciferase were determined using the dual-luciferase reporter assay system (Promega) according to manufacturer instructions. Assays were conducted the independent experiments at least in triplicate. The data represent relative Firefly luciferase activity normalized to Renilla luciferase activity.

### Statistical analysis

Data analyses were performed as mean ± SD. Differences in each group were examined for statistical significance using Student’s *t*-test and *P* values less than 0.05 was considered statistically significant.

### Data availability

The authors confirm that all data underlying the findings are fully available without restriction. All relevant data are within the paper.
